# Author Correction: Age-related epithelial defects limit thymic function and regeneration

**DOI:** 10.1038/s41590-026-02489-4

**Published:** 2026-03-13

**Authors:** Anastasia I. Kousa, Lorenz Jahn, Kelin Zhao, Angel E. Flores, Dante Acenas, Emma Lederer, Kimon V. Argyropoulos, Andri L. Lemarquis, David Granadier, Kirsten Cooper, Michael D’Andrea, Julie M. Sheridan, Jennifer Tsai, Lisa Sikkema, Amina Lazrak, Katherine Nichols, Nichole Lee, Romina Ghale, Florent Malard, Hana Andrlova, Enrico Velardi, Salma Youssef, Marina Burgos da Silva, Melissa Docampo, Roshan Sharma, Linas Mazutis, Verena C. Wimmer, Kelly L. Rogers, Susan DeWolf, Brianna Gipson, Antonio L. C. Gomes, Manu Setty, Dana Pe’er, Laura Hale, Nancy R. Manley, Daniel H. D. Gray, Marcel R. M. van den Brink, Jarrod A. Dudakov

**Affiliations:** 1 Program in Immunology, Memorial Sloan Kettering Cancer Center, New York, NY USA; 2 Translational Science and Therapeutics Division, and Immunotherapy Integrated Research Center, Fred Hutchinson Cancer Center, Seattle, WA USA; 3 City of Hope Los Angeles and National Medical Center, Duarte, CA USA; 4 The Walter and Eliza Hall Institute of Medical Research, Parkville, Victoria Australia; 5 Department of Medical Biology, The University of Melbourne, Parkville, Victoria Australia; 6 Department of Genetics, University of Georgia, Athens, GA USA; 7 Department of Immunology, University of Washington, Seattle, WA USA; 8 Department of Pathology and Laboratory Medicine, Memorial Sloan Kettering Cancer Center, New York, NY USA; 9 Computational and Systems Biology Program, Memorial Sloan Kettering Cancer Center, New York, NY USA; 10 Institute of Computational Biology, Helmholtz Center Munich, Munich, Germany; 11 Sorbonne Université, Centre de Recherche Saint-Antoine INSERM UMRs938, Service d’Hématologie Clinique et de Thérapie Cellulaire, Hôpital Saint Antoine, AP-HP, Paris, France; 12 Division of Pediatric Hematology and Oncology, Bambino Gesù Children’s Hospital, IRCCS, Rome, Italy; 13 Department of Medicine, Memorial Sloan Kettering Cancer Center, New York, NY USA; 14 Basic Sciences Division & Translational Data Science Integrated Research Center, Fred Hutchinson Cancer Center, Seattle, WA USA; 15 Human Vaccine Institute, Duke University, Durham, NC USA; 16 School of Life Sciences, Arizona State University, Phoenix, AZ USA

**Keywords:** Thymus, Bone marrow transplantation, Lymphopoiesis

Correction to: *Nature Immunology* 10.1038/s41590-024-01915-9, published online 7 August 2024.

In the version of this article initially published, in Extended Data Fig. 7a, due to a figure assembly mistake, the images shown as “Mouse 1, 2, 3” on the right-hand panel were mistakenly images representing regions 1–3 from one representative mouse. The images have been replaced with correct data from three different aged reporter mice. The figure is updated in the HTML and PDF versions of the article. For comparison, the original figure is available as Supplementary Information alongside this amendment.

## Supplementary information

Original, uncorrected Extended Data Fig. 7

