# Supplementary material for: Age-related epithelial defects limit thymic function and regeneration
Source: Nat Immunol. 2024 Aug 7;25(9):1593–606. doi: 10.1038/s41590-024-01915-9 (PMC11362016; doi:10.1038/s41590-024-01915-9)
Supplement: Supplementary file 1 — Supplementary Figs. 1 and 2, Table of gating for flow cytometry and Antibody reagent table. [file 41590_2024_1915_MOESM1_ESM.pdf]

nature immunology

Supplementary information

https://doi.org/10.1038/s41590-024-01915-9

Age-related epithelial defects limit thymic function and regeneration

In the format provided by the authors and unedited

Supplementary Figure 1

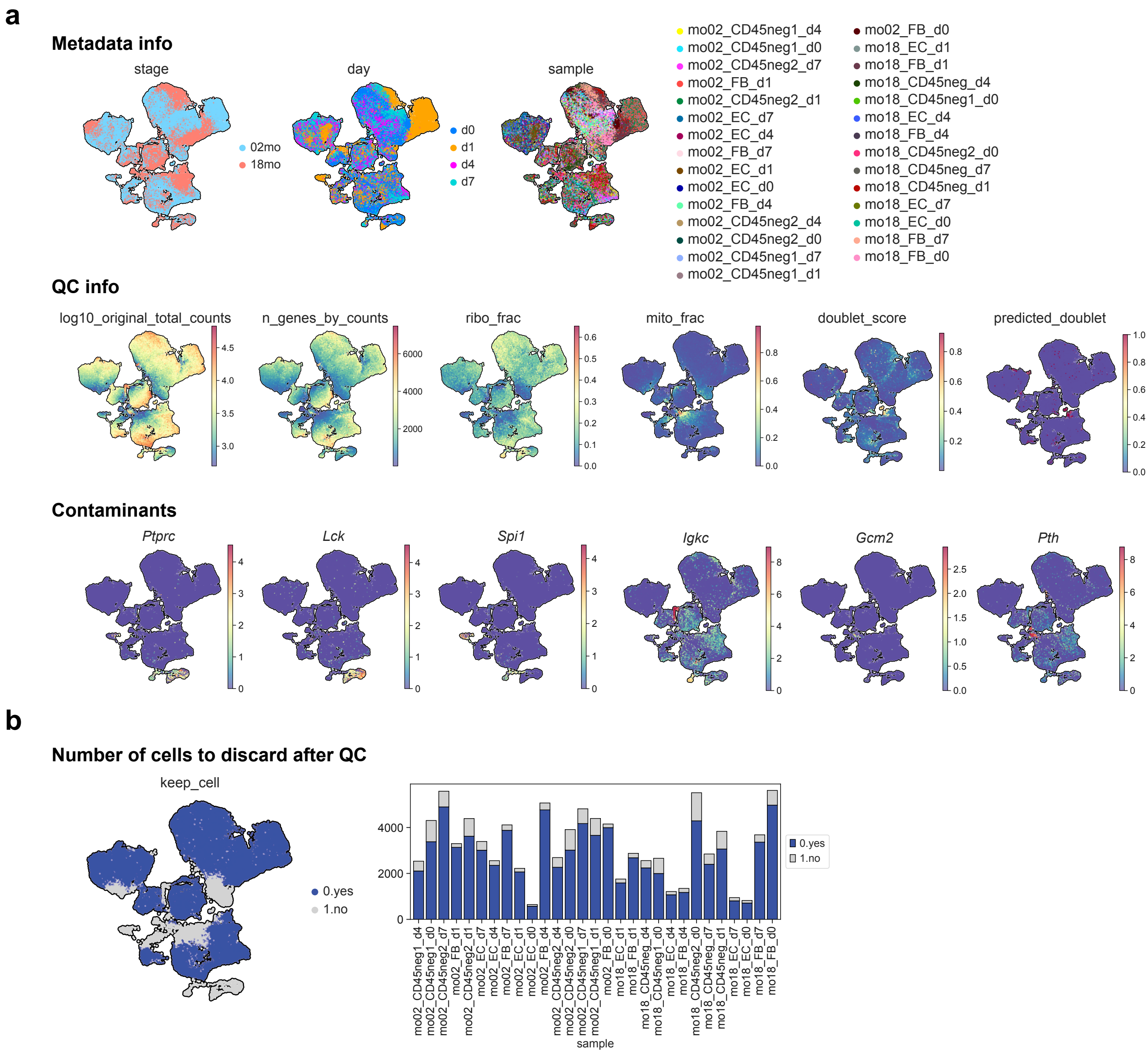

Supplementary Figure 1: **a,** UMAPs of 93,738 CD45- cells from 2mo or 18mo thymus at baseline (day 0) and at days 1, 4 and 7 after TBI prior to quality control. UMAPs in sequence display age cohort, day after TBI and origin sample (Metadata info); total counts in log10 scale, number of genes, ribosomal fraction, and mitochondrial fraction per cell (QC info); CD45+ and parathyroid contaminating cells expressing Ptprc, Lck, Spi1, Igkc and Gcm2, Pth genes respectively (Contaminants). **b,** UMAP and associated stacked barplot representation of filtered out cells on a per sample basis. Cells retained for further processing are shown in blue, while cells removed are shown in grey.

Supplementary Figure 2

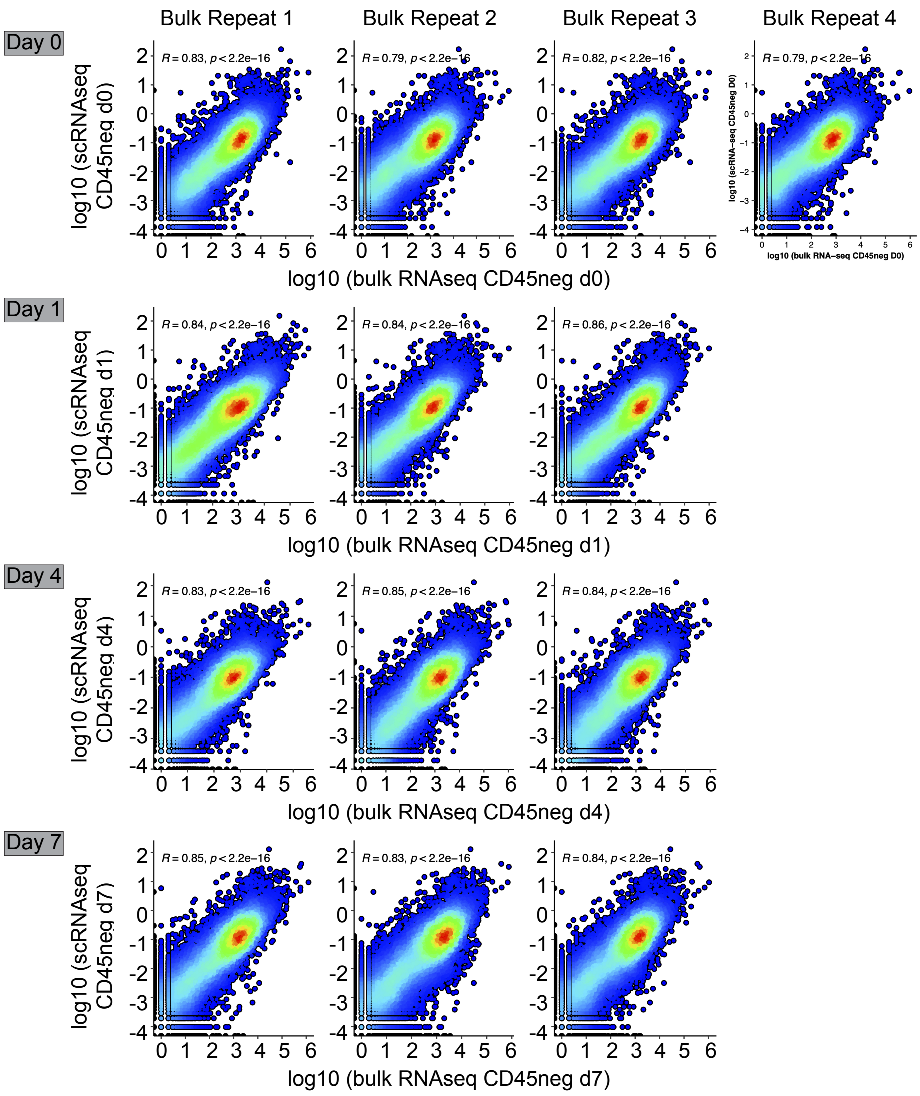

Supplementary Figure 2: Scatterplots showing correlation (Pearson) between averaged expression profiles of CD45- scRNA-seq averaged technical replicates collected at steady state (row 1), day 1 (row 2), day 4 (row 3) and day 7 (row 4) after TBI versus the expression profiles of CD45- bulk RNA-seq biological replicates (replicate 1; column 1, replicate 2; column 2, replicate 3; column 3, replicate 4; column 4) obtained on the same timeframe.

Supplementary Table 11: Gating strategy for cell subset analysis by flow cytometry

Subset	Flow phenotype	Figure
EC	LiveTER119$^-$CD45$^-$CD31$^+$PDGFRa$^-$	Ext Fig. 1
Fibroblast	LiveTER119$^-$CD45$^-$CD31$^-$PDGFRa$^+$	
mTEC	LiveTER119$^-$CD45$^-$EpCAM$^+$MHCII$^+$UEA1^{hi}Ly51^{lo}	
cTEC	LiveTER119$^-$CD45$^-$EpCAM$^+$MHCII$^+$UEA1^{lo}Ly51^{hi}	
mTEC1	LiveTER119$^-$CD45$^-$EpCAM$^+$MHCII^{lo}UEA1^{hi}Ly51^{lo}L1CAM$^-$CD104$^+$	Figure 1
mTEC2	LiveTER119$^-$CD45$^-$EpCAM$^+$MHCII$^+$UEA1^{hi}Ly51^{lo}L1CAM$^-$CD104-Ly6D$^-$	
mTEC3	LiveTER119$^-$CD45$^-$EpCAM$^+$MHCII$^+$UEA1^{hi}Ly51^{lo}L1CAM$^-$CD104-Ly6D$^+$	
TEC^{tuft}	LiveTER119$^-$CD45$^-$EpCAM$^+$MHCII$^+$UEA1^{hi}Ly51^{lo}L1CAM$^+$	
DN-TEC	LiveTER119$^-$CD45$^-$EpCAM$^+$MHCII$^+$UEA1$^-$Ly51$^-$	
aaTEC1	LiveTER119$^-$CD45$^-$PDGFRa$^-$CD31$^-$EpCAM$^+$MHCII$^+$UEA1$^-$Ly51$^-$Claudin-3$^+$	Figure 1j, Ext Data Fig. 4
aaTEC2	LiveTER119$^-$CD45$^-$EpCAM$^-$MHCII$^+$PDPN$^+$PDGFRa$^-$	
capFB	LiveTER119$^-$CD45$^-$CD31$^-$PDGFRa$^+$CD26$^+$PDPN$^+$	Ext Data Fig. 4
medFB	LiveTER119$^-$CD45$^-$CD31$^-$PDGFRa$^+$CD26$^-$PDPN$^+$	
intFB	LiveTER119$^-$CD45$^-$CD31$^-$PDGFRa$^+$PDPN$^-$	
aEC	LiveTER119$^-$CD45$^-$CD31$^+$PDGFRa$^-$CD309$^-$CD62P$^-$	Ext Data Fig. 4
capEC	LiveTER119$^-$CD45$^-$CD31$^+$PDGFRa$^-$CD309$^+$	
vEC	LiveTER119$^-$CD45$^-$CD31$^+$PDGFRa$^-$CD309$^-$CD62P$^+$	
Pericyte	LiveTER119$^-$CD45$^-$CD31$^-$PDGFRb$^+$Ly51$^+$CD9$^+$	Ext Data Fig. 4
vSMC	LiveTER119$^-$CD45$^-$CD31$^-$PDGFRb$^+$Ly51$^-$CD9$^+$	
Mesothelial	LiveTER119$^-$CD45$^-$CD31$^-$PDGFRb$^-$CD9$^+$PDPN$^+$	
Tuft Cell	LiveTER119$^-$CD45$^-$EpCAM$^+$ UEA1^{hi}Ly51^{lo}GFP$^+$DCLK1$^+$Ly6D$^{lo/-}$	Ext Data Fig. 7 (In Foxn1^{nTnG} mice)
Corneocyte	LiveTER119$^-$CD45$^-$EpCAM$^+$ UEA1^{hi}Ly51^{lo}GFP$^+$DCLK1-Ly6D$^+$	
M cell	LiveTER119$^-$CD45$^-$EpCAM$^+$ UEA1^{hi}Ly51^{lo}GFP$^+$GP2$^+$Ly6D$^{lo/-}$	

Supplementary Table 12: Flow cytometry and cell sorting antibodies:

Antigen	Clone	Fluorochrome	Vendor	Cat#	Lot#	Dilution
CD45	30-F11	PercP-Cy5.5	Biolegend	103132	B363634	1/100
		PE-CF594	BD	562420	0013784	1/300
		BUV615	BD	751170		1/300
CD31	390	PercP-Cy5.5	Biolegend	102420	B247171	1/1300
TER119	TER-119	PercP-Cy5.5	Biolegend	116228	B356105	1/100
		BUV563	BD	741257	2265032	1/500
		PE-Cy5.5	Invitrogen	35-5921-82		1/650
MHCII	M5/114.15.2	BV421	Biolegend	107631	B360795	1/300
		BV711	Biolegend	107631	B377900	1/400
		APC-e780	Invitrogen	47-5321-82		1/2000
EpCAM	G8.8	APC-Cy7	Biolegend	118218	B393333	1/100
		Percp-e710	Invitrogen	46-5791-82	4278076	1/400
Ly51	6C3	PE-Cy7	Biolegend	108313	B215053	1/100
		PE	Invitrogen	12-5891-83	4300066	1/1000
		Alexa 647	Biolegend	108312	B222435	1/400
PDGFRa	APA5	BUV737	BD	741789	3228775	1/200
		Alexa594	Invitrogen	14-1401-82		1/100
		BV750	BD	747220		1/100
PDGFRb	APB5	SB600	Invitrogen	63-1402-80		1/100
CD104	346-11A	PE-Cy7	Biolegend	123615	B343426	1/200
		BV711	BD	743082		1/150
L1CAM	555	PE	RD system	AAYQ0223061	FAB5674P	1/100
Ly6D	49-H4	eFluor450	eBio	48-597480	2020836	1/200
PDPN	8.1.1	APC-Fire750	Biolegend	127425	B313124	1/200
		BUV805	BD (custom)	624287		1/650
		Purified	Biolegend	127402	B219285	1/100
	Alexa 647	Anti-IgG	Invitrogen	A-21451	2527971	1/100

CD26	H194-112	BUV737	BD	741729		1/20
CD62P	RB40.34	BV480	BD	746249		1/300
CD309	Avas12a	BUV661	BD	741571		1/50
CD9	KMC8	BUV563	BD	741270		1/50
Claudin-3<br>Anti-Rabbit	Polyclonal<br>Secondary	Purified<br>APC	Invitrogen<br>Invitrogen	34-1700<br>A31573	Y1376567<br>1786284	1/100<br>1/400
UEA1	Lectin	Fitc	VectorLabs	FL-1061	ZC0426	1/1000
		Biotin	VectorLabs	B1065	ZD0810	1/200
		Strep-BV605	Biolegend	405229	B387251	1/1000
pAKT	Polyclonal	Pac Blue	Cell Signaling	68035S	1	1/100
FOXN1	2/41	Alexa 647	Gift	N/A	N/A	
DCLK1	aa690-720	BV421	Lsbio	LSC100746	134142	1/1000
GP2	2F11-C3	Alexa 594	MBL	D278-3	010	1/100
GFP	GFP-1020	Dylight 488	Aves	GFP-1020	GFP3717982	1/1000

Supplementary Table 13: Antibodies for immunofluorescence.

	Antigen	Format	Vendor	Catalog#
Primaries	pan-Cytokeratin	Purified	Dako	Z0622
	Keratin 5	Purified	Biolegend	poly19055
	anti-mouse K8/18	Purified	Developmental Studies Hybridoma Bank	TROMA-1
	Keratin 14	Purified	Abcam	EPR17350
	AIRE	Purified	WEHI	Clone# 5H12
	DCLK1	Purified	LSBio	LS-C100746
	UEA1 lectin	Purified	Vector labs	B-1065
Secondaries	Donkey anti-rabbit IgG	Alexa Fluor® 647	Invitrogen	A31573
	Goat anti-rat IgG	Alexa Fluor® 647	Invitrogen	A-21247
	Streptavidin conjugate	Alexa Fluor® 647	Invitrogen	S21374
