# Supplementary material for: Author Correction: Age-related epithelial defects limit thymic function and regeneration
Source: Nat Immunol. 2026 Mar 13;27(5):1082. doi: 10.1038/s41590-026-02489-4 (PMC13132717; doi:10.1038/s41590-026-02489-4)
Supplement: Supplementary file 1 — Original, uncorrected Extended Data Fig. 7 [file 41590_2026_2489_MOESM1_ESM.pdf]

nature immunology

Supplementary information

Author Correction: Age-related epithelial defects limit thymic function and regeneration

In the format provided by the authors and unedited

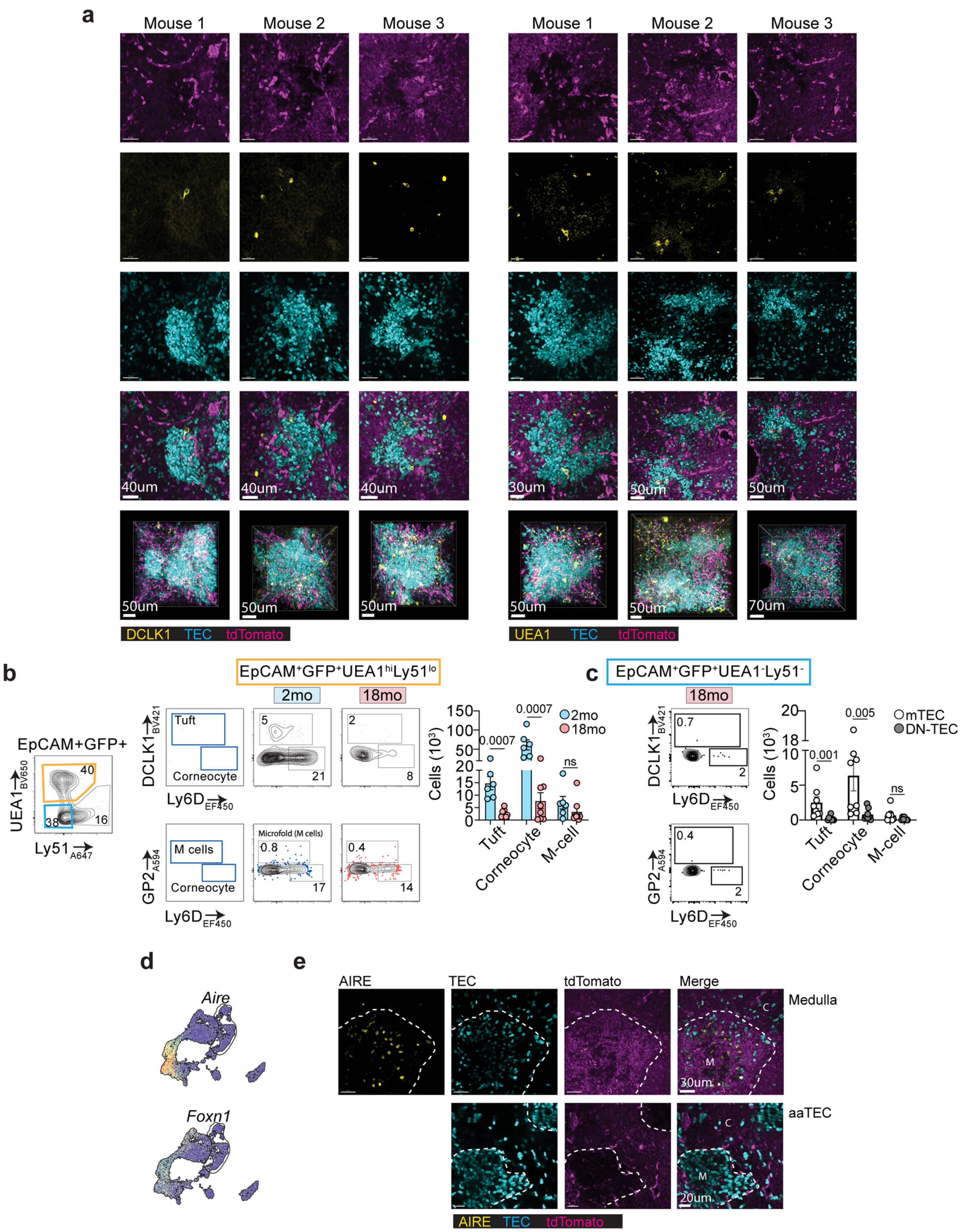

Extended Data Fig. 7
